# Transfer Learning Empowered Multiple‐Indicator Optimization Design for Terahertz Quasi‐Bound State in the Continuum Biosensors

**DOI:** 10.1002/advs.202504855

**Published:** 2025-04-27

**Authors:** Shengfeng Wang, Bingwei Liu, Xu Wu, Zuanming Jin, Yiming Zhu, Linjie Zhang, Yan Peng

**Affiliations:** ^1^ Shidong Hospital Affiliated to University of Shanghai for Science and Technology Terahertz Technology Innovation Research Institute Shanghai Key Lab of Modern Optical System Shanghai Institute of Intelligent Science and Technology University of Shanghai for Science and Technology 516 Jungong Road Shanghai Shanghai 200093 China; ^2^ State Key Laboratory of Quantum Optics and Quantum Optics Devices Institute of Laser Spectroscopy Shanxi University 92 Wucheng Road Taiyuan Shanxi 030006 China; ^3^ Shanghai Institute of Intelligent Science and Technology Tongji University 1239 Siping Road Shanghai Shanghai 200092 China

**Keywords:** inverse design, metasurface biosensor, multiple indicator optimization, terahertz, transfer learning

## Abstract

Terahertz metasurface biosensors based on the quasi‐bound state in the continuum (QBIC) offer label‐free, rapid, and ultrasensitive biomedical detection. Recent advances in deep learning facilitate efficient, fast, and customized design of such metasurfaces. However, prior approaches primarily establish one‐to‐one mappings between structure and optical response, neglecting the trade‐offs among key performance indicators. This study proposes a pioneering method leveraging transfer learning to optimize multiple indicators in metasurface biosensor design. For the first time, multiple‐indicator comprehensive optimization of the quality (Q) factor, figure of merit (FoM), and effective sensing area (ESA) is achieved. The two‐stage transfer learning method pre‐trains on low‐dimensional datasets to extract shared features, followed by fine‐tuning on complex, high‐dimensional tasks. By adopting frequency shift as a unified criterion, the contribution ratios of these indicators are quantified as 26.09% for the Q factor, 48.42% for FoM, and 25.49% for ESA. Compared to conventional deep‐learning approaches, the proposed method reduces data requirements by 50%. The biosensor designed using this method detects the biomarker homocysteine, achieving detection at the ng µL^−1^ level, with experimental results closely matching theoretical predictions. This work establishes a novel paradigm for metasurface biosensor design, paving the way for transformative advances in trace biological detection.

## Graphical abstract

This study proposes a pioneering method leveraging transfer learning to optimize multiple indicators in meta‐biosensor design, achieving the multiple‐indicator comprehensive optimization. By adopting frequency shift as a unified criterion, this study quantifies the contribution ratio of each performance indicator to the biosensor, which facilitates achieving a balance among multiple indicators to enhance the overall performance of the biosensor.

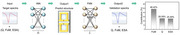

## 1 Introduction

Terahertz waves (0.1–10.0 THz) have demonstrated significant potential in the biomedical field due to their unique non‐ionizing properties, high penetration capability, and sensitivity to molecular vibrational modes.^[^ ^1^, ^2^, ^3^, ^4^, ^5^ ^]^ Especially in trace molecular detection, metasurface‐based biosensors have become ideal detection platforms due to advantages such as label‐free detection, fast response, and high sensitivity. Composed of artificial subwavelength‐scale structural units in two dimensions, metasurfaces greatly strengthen light‐matter interactions by precisely controlling the phase, amplitude, and polarization of electromagnetic waves.^[^ ^6^, ^7^, ^8^, ^9^, ^10^, ^11^, ^12^, ^13^ ^]^ Metasurface devices, especially those based on the quasi‐bound state in the continuum (QBIC) mechanism, can achieve ultra‐high quality (Q) factor resonance, enabling strong electromagnetic energy confinement within the structure.^[^ ^14^, ^15^, ^16^, ^17^, ^18^, ^19^, ^20^, ^21^ ^]^ This significantly enhances the signal response of the sample, offering a means to overcome the detection limits for trace biological molecules. Currently, the terahertz metasurface biosensor (meta‐biosensor) has been widely applied to thecells, proteins, and viruses, among others.^[^ ^22^, ^23^, ^24^, ^25^, ^26^, ^27^, ^28^, ^29^ ^]^ However, as related research progresses, several critical issues in the sensor design process still need to be addressed.

Traditional metasurface design methods typically rely on complex electromagnetic simulations and finite element methods. These methods search for optimal solutions through extensive parameter sweeps, resulting in high computational costs and extended design cycles.^[^ ^30^, ^31^, ^32^ ^]^ With the rapid development of machine learning technology,^[^ ^33^, ^34^, ^35^, ^36^, ^37^, ^38^, ^39^, ^40^, ^41^ ^]^ deep learning‐based design methods have been proposed to predict the mapping relationships between sensor structures and spectral responses, significantly improving design efficiency.^[^ ^42^, ^43^, ^44^, ^45^, ^46^, ^47^, ^48^, ^49^ ^]^ Integrating deep learning with physics in metasurface design has also been proposed as an effective approach to enhance the performance of deep learning models.^[^ ^50^, ^51^ ^]^ However, the operation of deep learning models typically relies on large, high‐quality datasets that are time‐consuming to acquire and rigorously validate.^[^ ^52^, ^53^, ^54^ ^]^ In particular, as research variables expand from a single parameter to multiple parameters, the dataset size required for deep learning grows exponentially. This not only exacerbates the challenges of data acquisition and validation but also directly constrains the efficiency of deep learning‐based design. To enhance model performance under data‐limited conditions, transfer learning has been proposed and shown to be highly effective.^[^ ^55^, ^56^, ^57^ ^]^ By transferring the knowledge from pre‐trained models to new tasks, transfer learning can maintain the modeling capabilities of deep learning while significantly reducing data dependencies.^[^ ^58^, ^59^, ^60^, ^61^ ^]^ However, in the design of terahertz meta‐biosensors, transfer learning has not yet been effectively applied due to an incomplete parameter evaluation system.

Current meta‐biosensor designs focus on optimizing a single performance indicator, such as the Q factor,^[^ ^62^, ^63^, ^64^ ^]^ figure of merit (FoM),^[^ ^65^, ^66^ ^]^ or effective sensor area (ESA).^[^ ^67^ ^]^ However, in practical biosensing, the overall performance of the biosensor is not determined by a single indicator, but by the combined effect of multiple performance indicators. This presents significant technical challenges in the optimization design of meta‐biosensors. Firstly, complex nonlinear coupling relationships exist between different performance indicators. Optimizing one indicator in isolation can inadvertently diminish others. For example, excessive Q factor enhancement may lead to reductions in both FoM and ESA, thus reducing the overall sensitivity of the sensor. This nonlinear coupling between multiple objectives requires balancing the interactions among all relevant indicators, aiming for a global optimum rather than merely maximizing a single indicator. Furthermore, the structural design of biosensors typically involves multiple geometric parameters, and the number of parameter combinations increases exponentially with each additional variable, which significantly increases the complexity of multi‐indicator optimization and design. This not only increases computational demands but also necessitates the use of advanced optimization algorithms to effectively explore the complex design space. Under conditions of limited data, extracting effective design rules and achieving precise predictions remains a critical issue to be addressed in multi‐indicator optimization. As a result, developing strategies for collaborative multi‐indicator optimization and quantifying the contribution of each performance indicator to biosensor performance are key challenges that must be addressed in the design of meta‐biosensors.

In this work, we propose a novel method that captures shared patterns in the data by leveraging pre‐training on low‐dimensional datasets and applying transfer learning to uncover the mapping relationships in high‐dimensional datasets. Using frequency shift as the unified performance evaluation criterion, our approach enables the collaborative optimization of multiple critical indicators (Q factor, FoM, and ESA) in meta‐biosensors, elucidating their contributions to device performance and achieving an optimized terahertz meta‐biosensor design. Compared with traditional finite element simulations, this method enhances computational efficiency by nearly four orders of magnitude while reducing data requirements by approximately 50% compared to conventional deep learning approaches. By defining the Q factor, FoM, and ESA as key neural network outputs and employing frequency shift as the comprehensive biosensor performance evaluation indicator, we successfully achieved the multi‐indicator collaborative optimization and quantified the contribution ratio of each indicator on biosensing performance. This finding provides a crucial theoretical foundation for optimization design. The meta‐biosensor designed through this method exhibits strong consistency between experimental results and theoretical predictions, enabling trace‐level detection of biomarkers. Beyond enabling efficient trace biological molecule detection, this approach opens a new research direction for multi‐indicator collaborative optimization in biosensors, laying a critical foundation for the design and development of intelligent optical devices.

## 2 Result and Discussion

**Figure 1:**
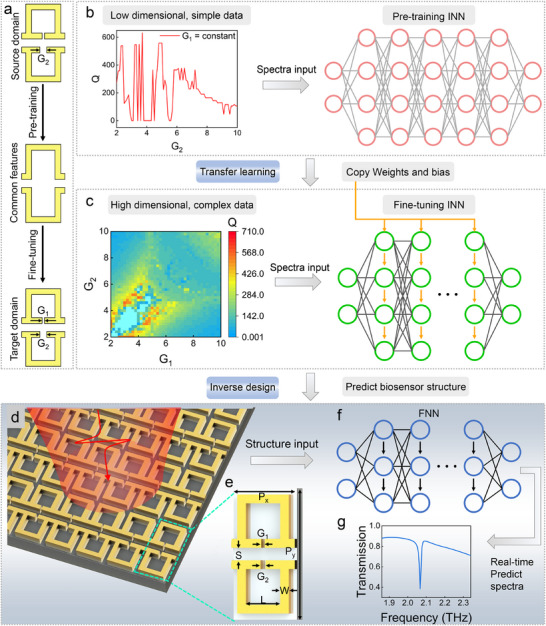
Design workflow of a meta‐biosensor based on transfer learning and multi‐objective collaborative optimization. a) Schematic of the transfer learning framework, consisting of two core modules: pre‐training and fine‐tuning. b) Overview of the pre‐training process, where low‐dimensional simple data are used for initial model training. c) Overview of the fine‐tuning process, where the pre‐trained model is optimized using high‐dimensional complex data to meet specific application requirements. d) The meta‐biosensor was designed through multi‐indicator collaborative optimization. e) Geometric parameters of the unit lattice in the designed meta‐biosensor. f) FNN model for real‐time verification of the designed structure. g) Real‐time prediction of the spectral response of the designed meta‐biosensor by the model.

This study introduces a transfer‐learning‐based inverse design method for meta‐biosensors, aimed at collaboratively optimizing multiple performance indicators. Initially, we pre‐trained the model on simple low‐dimensional data to capture general design principles (**Figure** **1**b). The knowledge gained during the pre‐training phase serves as an effective initial condition for designing more complex metasurfaces, accelerating the design process and improving accuracy. In the transfer learning phase, the model balances the pre‐trained knowledge with the new task requirements through a fine‐tuning strategy (Figure 1c). While designing the meta‐biosensor structure using INN, our model simultaneously predicts its spectral response in real‐time with FNN (Figure 1d–g). Leveraging this model, we successfully optimized the design of the meta‐biosensor, achieving the collaborative optimization of the Q factor, FoM, and ESA.

### 2.1 Design and Performance Analysis of the Pre‐Training Network Model

In the pre‐training phase, we adopted a tandem neural network architecture that combines forward neural networks (FNN) and inverse neural networks (INN), which achieves a closed‐loop mapping between the spectral response and the geometric structure. This design effectively alleviates the non‐uniqueness problem in the inverse design of electromagnetic wave scattering, providing a reliable modeling foundation for transfer learning. Specifically, the INN takes target spectra and performance indicators (Q factor, FoM, ESA) as input and predicts the corresponding geometric parameters (G_1_ and G_2_) of the meta‐biosensor structure. This enables the rapid generation of candidate structural designs that meet specific spectra and performance indicator requirements, significantly improving the efficiency of the inverse design process. The FNN is trained to predict the transmission spectrum and performance indicators from the structural parameters. It is used to validate whether the designs generated by the INN produce spectra responses and performance indicators consistent with the original targets, thereby ensuring the physical feasibility of the inverse predictions. Its input consists of the output from INN, and its output is the spectral transmittance and performance indicators. During the multi‐indicator optimization stage, the FNN serves as a fast and accurate forward predictor, enabling the rapid evaluation of structures with significantly reduced computational cost compared to electromagnetic simulations such as COMSOL. Notably, although the INN is not directly involved in this final optimization step, its joint training with the FNN ensures that the inverse predictions are constrained by and consistent with realistic forward responses. The tandem of the INN model and the FNN model mitigates the effects of non‐unique solutions on the model and enhances the robustness and physical validity.

**Figure 2:**
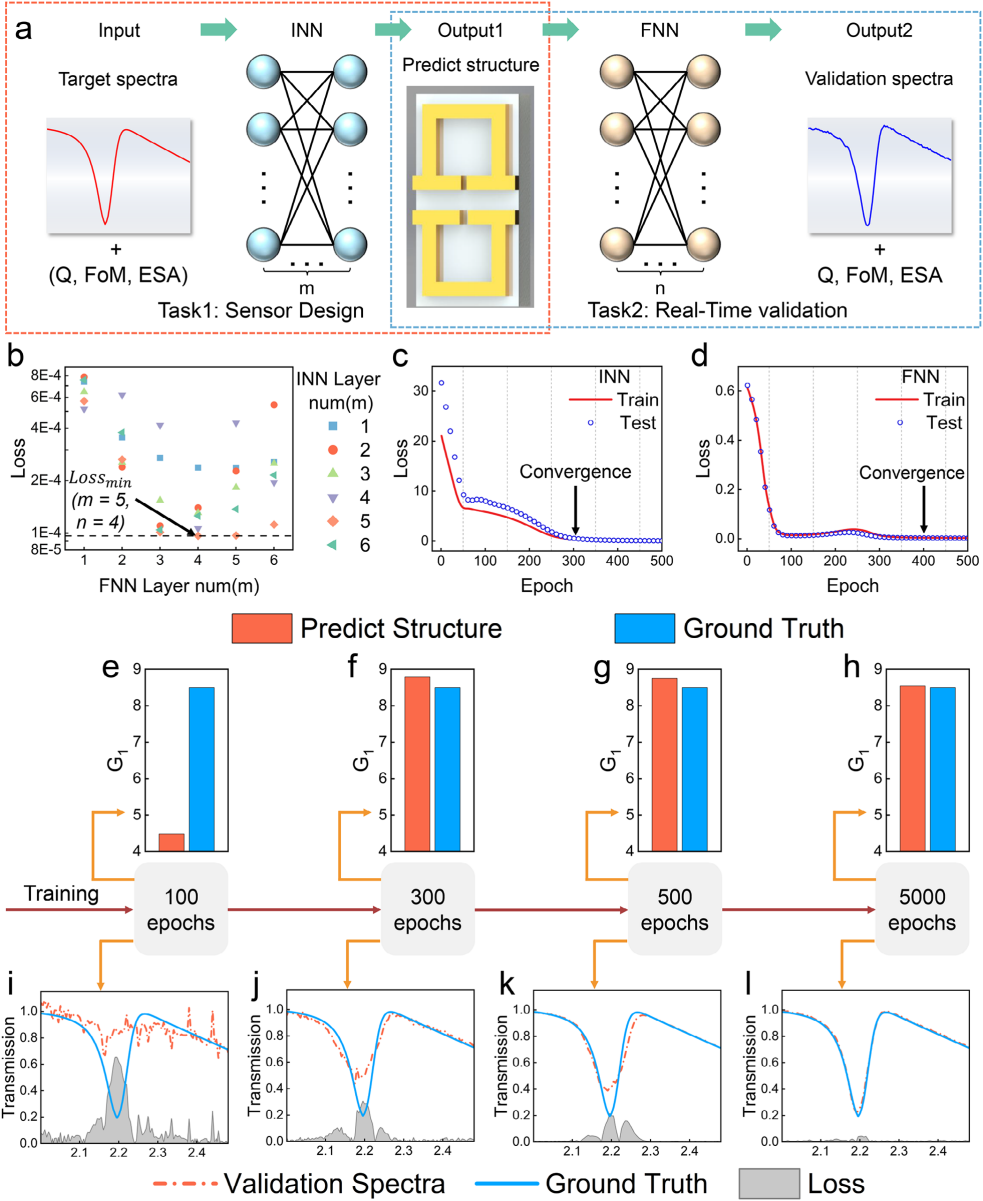
Pretraining network model. a) Structure of the tandem neural network, consisting of a Forward Neural Network and an Inverse Neural Network in series. b) Impact of the number of hidden layers on the network model error. c, d) Loss curves of the FNN and INN on the training and validation datasets. e—h) Differences between the design structure and the actual structure of the INN after 100, 300, 500, and 5000 training iterations. i–l) Differences between the reconstructed spectrum from the FNN and the COMSOL simulation spectrum after 100, 300, 500, and 5000 training iterations.

During model design, we systematically evaluated the impact of hidden layer count on performance. To determine the optimal configuration, we analyzed the mean squared error (MSE) of the model across various hidden layer settings during training (**Figure** **2**b). Our findings reveal that the lowest validation error (MSE = 0.0082) is achieved when the INN comprises five hidden layers and the FNN comprises four. Increasing the number of hidden layers allows the network to capture nonlinear features in high‐dimensional data more effectively. However, exceeding a certain threshold diminishes generalization, primarily due to overfitting and gradient vanishing. Further analysis of model error shows that the INN error converges after approximately 400 epochs, with only marginal improvement thereafter (Figure 2c). For the FNN, after 200 epochs, the errors in both training and test sets show a slight increase, likely caused by noise. Nevertheless, with continued training, the model balances noise interference and data patterns, resulting in error convergence after 400 epochs (Figure 2d).

We further evaluated the model design capability and spectral reconstruction ability (see Figure S1, Supporting Information for details). A set of metasurface structures and their corresponding spectral responses were randomly selected from the validation dataset to assess model performance. As training iterations progressed, the structural parameters predicted by INN gradually converged toward the true values, with the error dropping from 47.36% after 100 iterations to just 0.5% after 5000 iterations (Figure 2e–h). In these figures, red bars represent INN‐predicted parameters, while blue bars denote actual structural parameters. This indicates that the INN model effectively learns the mapping between the spectral and the structural parameters, achieving optimal solutions rapidly. Likewise, the FNN exhibited strong spectral reconstruction capability (Figure 2i–l). The red dashed line represents the FNN‐predicted spectra, the blue solid line shows the simulation results, and the gray shaded area indicates prediction errors for absorption responses at different frequencies. After 500 iterations, FNN‐predicted spectra closely matched the simulated spectra, indicating excellent performance in predicting the spectral response of designed structures.

### 2.2 Advancing Model Performance by Transfer Learning Strategy

**Figure 3:**
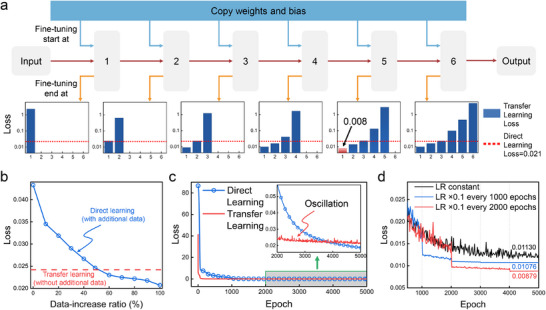
Fine‐tuning network model. a) The effect of fine‐tuning each layer on model error. b) Performance comparison between the transfer learning model and the tandem network model with datasets of different sizes. c) Loss curves of the transfer learning model and the tandem network model. d) Comparison of performance for the transfer learning model with different learning rate adjustment strategies.

We conducted a comprehensive analysis to evaluate the impact of various fine‐tuning strategies on model performance. Initially, we explored the effect of selectively freezing and fine‐tuning specific layers within the transfer learning model (**Figure** **3**a). The six graphs illustrate different model architectures, where the x‐axis indicates the starting layer for fine‐tuning, the y‐axis denotes the model error, and each graph corresponds to fine‐tuning up to layers 1 through 6. The results indicate that freezing the sixth layer while fine‐tuning the first five layers minimizes the model loss to 0.008. These findings underscore the importance of strategically selecting fine‐tuning layers within the transfer learning framework to optimize model performance effectively.

Next, we examined the impact of dataset size on model performance by comparing transfer learning with conventional deep learning models. The comparison results reveal that the transfer learning model significantly reduces the demand for large‐scale datasets, which is about 50% lower than that of the deep learning model (Figure 3b). The blue dotted line represents the error of the deep learning model with additional dataset support, while the red dashed line shows the error of the transfer learning model without additional dataset support. Especially in data‐limited scenarios, the transfer learning model achieves superior performance, maintaining a low error of 0.024 without additional data, compared to 0.043 for the deep learning model. This advantage stems from the pre‐training phase of transfer learning, where common features are extracted from simple low‐dimensional tasks, significantly enhancing the model generalization capacity. In contrast, deep learning models trained directly require significantly more data to achieve comparable performance. Even when the deep learning model is trained with an additional 50% of the dataset, its error remains at 0.025, still higher than the transfer learning model. Only when the deep learning model is provided with an additional 100% of the dataset does its error drop to 0.021. This is because the large‐scale datasets can better learn the intrinsic features and patterns in the data, thus improving the model generalization ability.

Finally, we assessed the effectiveness of different learning rate adjustment strategies in optimizing model performance. We compared the performance of the transfer learning model (red curve) with a deep learning model trained with a 100% additional dataset (blue curve) during the training process (Figure 3c). As shown in the figure, during the early training stages, the transfer learning model demonstrates a lower error rate and faster convergence due to the knowledge retained from the pre‐trained network that helps the model adapt to the new task more quickly. However, in the later training stages (as highlighted in the inset showing epochs 2000 to 5000), the transfer learning model exhibits error oscillations, occasionally exceeding the error levels of the deep learning model. This instability is typically attributed to an excessively high learning rate.

To overcome this issue, we evaluated three distinct learning rate adjustment strategies for the transfer learning model: (1) maintaining a constant learning rate, (2) reducing the learning rate to 10% every 1000 epochs, (3) reducing the learning rate to 10% every 2000 epochs. Dynamic learning rate adjustments allow for an initially high rate to accelerate parameter updates, followed by a gradual reduction to refine optimization near local optima, which enhances overall performance. The choice of learning rate adjustment strategy has a significant impact on the transfer learning model error (Figure 3d). The black curve represents the model with a constant learning rate, resulting in a final error of 0.0113. The red and blue curves correspond to models where the learning rate is reduced to 10% every 1000 and 2000 epochs, respectively. The results show that incorporating dynamic learning rate adjustments progressively reduces model error. Notably, the model with the learning rate reduced to 10% every 2000 epochs achieved a final error of 0.0087, outperforming the other strategies and significantly reducing oscillations during training.

To further evaluate the efficiency of our model, we compared it with traditional electromagnetic simulation methods. Conventional electromagnetic simulations take approximately 3600 seconds per design, whereas our approach completes predictions in just 0.1 seconds, representing a 36000‐fold improvement in efficiency. Moreover, with prediction errors below 1%, our model closely aligns with ground truth values, establishing a foundation for advancing multi‐indicator collaborative optimization design.

### 2.3 Optimization Design of Multiple Performance Indicators in Meta‐Biosensors

The performance of meta‐biosensors is primarily determined by three critical indicators: the Q factor, FoM, and ESA, each of which significantly influences biosensing effectiveness. The Q factor represents the quality of the biosensor, with higher values signifying superior resolution of indicator signals and lower energy loss. The FoM considers the Q factor and resonance intensity (I), where higher values reflect an optimal balance between signal resolution and strength. The ESA quantifies the detection range of the biosensor, with larger values increasing the probability of capturing target molecules, thereby improving detection efficiency. However, these indicators exhibit nonlinear coupling, optimizing one in isolation may adversely affect the others, potentially degrading overall sensor performance. Achieving an optimal balance among the Q factor, FoM, and ESA is essential for enhancing the overall performance of biosensors, enabling high‐sensitivity and low‐error detection of trace molecules. Therefore, determining the optimal trade‐off between these performance indicators remains a key challenge in sensor design.

**Figure 4:**
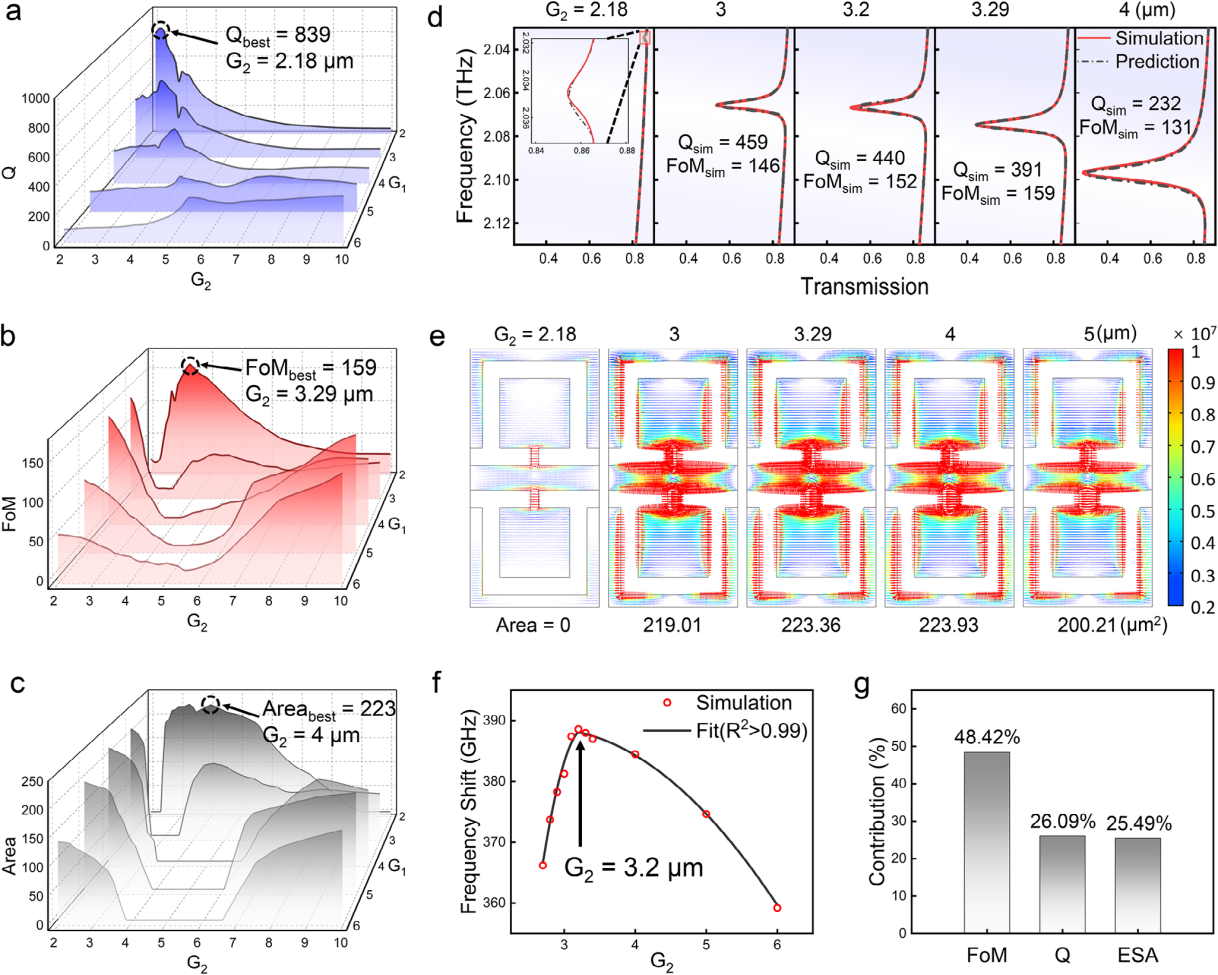
Collaborative optimization design and simulation verification of meta‐biosensor indicators. a–c) The Q factor, FoM, and ESA of meta‐biosensors with different structures were predicted by the transfer learning model. d) Comparison of network prediction and simulation results. e) The simulation results of the displacement electric field density distribution in the x‐y plane for different meta‐biosensors structures. f) With G_1_ fixed at 2 µm, the frequency shift of the simulated meta‐biosensor varies with the opening width G_2_. g) Analysis of the contribution of each evaluation indicator to sensor performance.

To address this challenge, we propose a transfer learning‐based methodology for optimizing multiple performance indicators in meta‐biosensors (see Figure S2, Supporting Information for details). First, our approach leverages transfer learning to predict and identify the range of meta‐biosensor structures that achieve high Q factor, FoM, and ESA. We analyzed the Q factor for different structural parameters using the transfer learning model (**Figure** **4**a). It can be observed that when G_1_ = 2 µm and G_2_ = 2.18 µm, the Q factor reaches its peak at 839.5623, and this structure significantly enhances the Q factor by strengthening the QBIC mechanism of the metasurface. We also employed the transfer learning model to predict the trend of FoM under different structural parameters (Figure 4b). When G_1_ = 2 µm and G_2_ = 3.29 µm, the FoM value reaches its maximum of 158.9113, achieving the best trade‐off between Q factor and resonance intensity. We also predicted the ESA of meta‐biosensors under different structural parameters (Figure 4c). When G_1_ = 2 µm and G_2_ = 4.01 µm, the ESA of the biosensor reaches its maximum value of 223.8061 µm^2^. When G_1_ = 2 µm and G_2_ = 3.29 µm, the ESA of the biosensor reaches 223.7581 µm^2^. These structures significantly enhance light‐matter interactions, expanding the detection range and increasing the probability of molecular interactions with the sensor, thereby enhancing the detection limit. This holds substantial importance for practical biosensing applications, especially in the detection of low‐concentration analytes. Compared to traditional simulation methods, our transfer learning model rapidly identifies the optimal solutions for the Q factor, FoM, and ESA, significantly reducing the design cycle. Through the rapid parameter search of the transfer learning model, we identified the structures that optimize each indicator. Subsequently, we determined that the structural range achieving the highest standards for Q factor, FoM, and ESA is G_1_ = 2 µm and G_2_ ranges from 2 µm to 4 µm. This provides a strong foundation for multi‐indicator optimization in biosensor design.

Next, we validated the key performance indicators predicted by the model through simulations to evaluate the prediction accuracy of the transfer learning model (see Figure S3, Supporting Information for details). We compared the predicted spectral responses of different structural biosensors with the corresponding simulation results (Figure 4d). The high consistency between model predictions and simulations confirms the high accuracy of the transfer learning model in optical performance prediction. Specifically, the network error for the Q factor is below 1.2%, and the FoM prediction error remains under 2%, further validating the reliability of the model. We also simulated and calculated the ESA of meta‐biosensors with different structures (Figure 4e). The analysis shows that when G_1_ = 2 µm and G_2_ = 4 µm, the ESA is 223.93 µm^2^, and when G_1_ = 2 µm and G_2_ = 3.2 µm, the ESA is 223.36 µm^2^. In both cases, the error between simulation and model prediction is less than 0.5%, confirming the accuracy and generalization ability of the transfer learning model in capturing complex structural performance. This strong consistency indicates that our transfer learning model can accurately predict multiple performance indicators even with limited data, effectively addressing the challenge of data scarcity in multi‐indicator collaborative optimization.

The Q factor, FoM, and ESA, as the core performance indicators of biosensors, evaluate biosensor performance in terms of resolution, signal response capability, and detection range, respectively. However, optimizing a single indicator in isolation cannot fully reflect the comprehensive response of biosensors in practical biological detection scenarios. Furthermore, the nonlinear interactions among these indicators complicate the optimization process. To overcome these limitations, it is essential to establish a unified evaluation criterion that holistically characterizes meta‐biosensor performance.

To better align with practical applications, we use the frequency shift induced by biological samples to represent the final meta‐biosensor performance (see Figure S4, Supporting Information for details). The frequency shift is calculated as Δ*f*  =  *f*(0) − *f*(*t*), where *f*(0) represents the resonance frequency of the bare metasurface, and *f*(*t*) is the resonance frequency after introducing a 5 µm biological sample. This metric directly reflects the response intensity to the target analyte of the biosensor, a larger frequency shift reflects better sensor performance. We simulated the frequency shift induced by adding biological samples as a function of G_2_ with G_1_ fixed at 2 µm (Figure 4f). The results indicate that when G_1_ = 2 µm and G_2_ = 3.2 µm, the meta‐biosensor achieves a maximum frequency shift of 388.6 GHz while comprehensively optimizing the Q factor, FoM, and ESA. For example, compared to the structure with G_1_ = 2 µm and G_2_ = 4 µm, this optimal structure exhibits a slightly lower ESA (223.36 µm^2^ vs 223.93 µm^2^). However, its higher Q factor and FoM values result in a larger frequency shift during sensing, demonstrating enhanced overall performance. Similarly, compared to the G_1_ = 2 µm and G_2_ = 3 µm structure, the optimal structure achieves better frequency shift performance due to its higher FoM and ESA values, despite a slight decrease in the Q factor. By employing the frequency shift as the unified evaluation criterion, we achieved the collaborative optimization of the Q factor, FoM, and ESA, confirming that the optimal biosensor structure is obtained with G_1_ = 2 µm and G_2_ = 3.2 µm. This comprehensive optimization not only enhances the overall performance of biosensors but also lays a robust foundation for practical applications and future research.

In the process of optimizing multiple performance indicators, we observed that different structures exhibit significant variations in the Q factor, FoM, and ESA. While some structures attain optimal values for one or more of these indicators, their corresponding final frequency shifts did not exhibit the best performance. Therefore, clarifying the specific contributions of Q factor, FoM, and ESA to sensor performance will assist in guiding the collaborative optimization of multiple indicators, ensuring an optimal balance among them.

To quantify the specific contributions of each indicator to the frequency shift, we employed the Random Forest method.^[^ ^68^, ^69^ ^]^ Random Forest is an ensemble learning algorithm based on decision trees that calculates feature importance scores by measuring the reduction in impurity at each decision tree split node. (see Figure S5, Supporting Information for details). We used the random forest method to quantitatively analyze the contributions of these three indicators to frequency shift by constructing datasets, training models, and analyzing feature importance (Figure 4g). First, we constructed the training dataset by using Q factor, FoM, and ESA as input features and frequency shift as the target variable, and applied the random forest model for training. Next, we calculated the contribution of each feature to impurity reduction at decision tree split nodes to evaluate their relative importance on frequency shift. The analysis reveals that the contribution of FoM, Q factor, and ESA on biosensor frequency shift is 48.42%, 26.09%, and 25.49%, respectively. Unlike traditional single‐indicator evaluation methods, we quantitatively analyzed the relative contributions of these three indicators in determining biosensor performance for the first time, thereby providing a solid theoretical foundation for multi‐indicator optimization. This enables designers to achieve an optimal balance among multiple performance indicators, thereby maximizing the overall performance of the meta‐biosensor. Our findings confirm that achieving global performance optimization in meta‐biosensors requires a balanced consideration of the Q factor, FoM, and ESA, ensuring that the biosensor performance is optimally coordinated.

The contribution ratio analysis is not intended as a fixed conclusion for all metasurface biosensor designs but rather serves as a general framework and methodology applicable to diverse device structures. These ratios are structure‐dependent and may vary with design parameters or operational conditions. Nevertheless, this approach offers both theoretical insight and practical value for multi‐indicator optimization. By identifying which performance metric is dominant in a given structure, designers can adjust the optimization direction accordingly. This reduces reliance on blind trial‐and‐error, narrows the parameter space, lowers computational cost, and enables more targeted and efficient optimization strategies across various biosensing scenarios. Overall, contribution ratio analysis enhances both the design process and the final performance of meta‐biosensors (see Figure S6, Supporting Information for details).

### 2.4 The Measurement of the Trace Molecular Sensing Using the Designed Meta‐Biosensor

**Figure 5:**
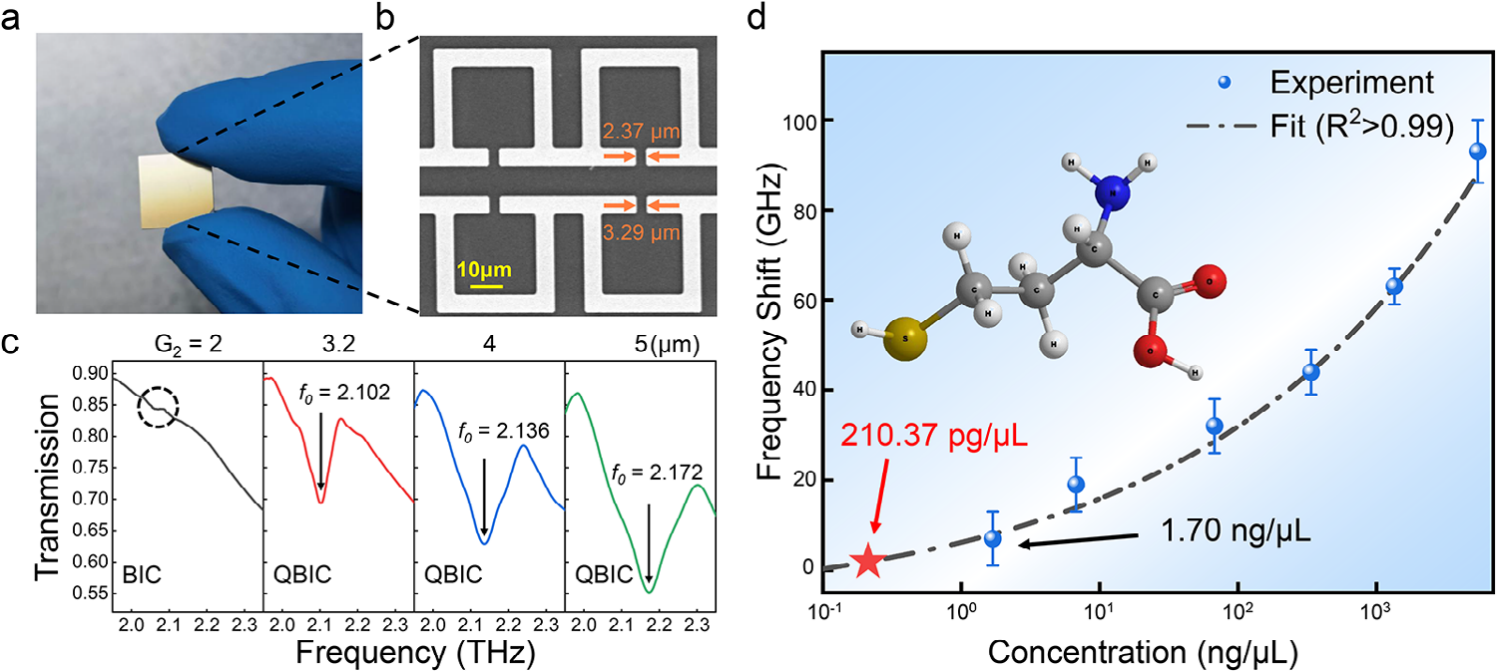
Perform performance tests on the metasurface designed by our model. a) The fabricated meta‐biosensor. b) SEM micrograph (Scale bar = 10 µm). c) The experimental spectral response of the fabricated meta‐biosensors with different structures. d) frequency shift as a function of biomarker concentrations, and the illustration is the molecular formula of homocysteine.

To validate the prediction accuracy of our method, we fabricated metasurface structures based on the optimized design parameters. We used conventional photolithography for fabrication (see Figure S7, Supporting Information for details). The optimal design parameters for the biosensor based on our method were G_1_ = 2.00 µm and G_2_ = 3.20 µm. However, due to limitations in fabrication precision, the actual fabricated parameters were G_1_ = 2.37 µm and G_2_ = 3.29 µm. The details of the designed biosensor are visualized through optical images and scanning electron microscope (SEM) images (**Figure** **5**a,b). For comparison, we also fabricated a series of meta‐biosensors with G_1_ fixed at 2.00 µm while varying G_2_ at 2.00 µm, 4.00 µm, and 5.00 µm. We then analyzed the transmission spectra of the fabricated biosensors to evaluate the practical feasibility of the optimized designs (Figure 5c). As the structural gap G_2_ increases, a slight frequency shift and broadening of the corresponding QBIC resonance peaks occur. This is due to the increase in structural asymmetry caused by the change in opening width, leading to the originally symmetry‐protected BIC state transitioning to a QBIC state with finite leakage. Notably, the meta‑biosensor with fabricated parameters G_1_ = 2.37 µm and G_2_ = 3.29 µm exhibits a Q factor of 58 and a FoM of 8.12, indicating superior device performance. In contrast, when G_2_ is set at 4.00 µm and 5.00 µm, the Q factor drops to 22 and 16, respectively, and the FoM decreases to 3.74 and 2.72, with a gradual decline in performance, consistent with the theoretical expectations. In addition, we analyzed the reasons for performance degradation (see Figure S8, Supporting Information for details).

Here, we selected homocysteine (Hcy) molecules as the target biological sample for detection (see Figure S9, Supporting Information for details). Homocysteine is a biomarker strongly associated with various diseases, such as cardiovascular diseases, aneurysms, and Parkinson's disease.^[^ ^70^, ^71^, ^72^ ^]^ Its detection at low concentrations is critical for the early diagnosis of these diseases. We applied a gradient of Hcy solutions corresponding to human concentration ranges onto the meta‐biosensor surface and measured the resulting frequency shift of the biosensor resonance peaks (Figure 5d). It can be observed that as the concentration of Hcy solution decreases from 5407.2 ng µL^−1^ to 1.7 ng µL^−1^, the frequency shift of the biosensor resonance peak gradually reduces. Due to the limited resolution of the instrument (7.6 GHz), the minimum detectable concentration corresponding to this resolution on the chip is 1.7 ng µL^−1^. Furthermore, considering that the optimal resolution of the commercial terahertz spectrometer we used is 1.9 GHz after iteration, we fitted the frequency shift versus concentration curve. Based on this analysis, we estimate that at a 1.9 GHz resolution, pg‐level trace molecule detection (210.37 pg µL^−1^) can be achieved, which could effectively support the disease diagnosis and treatment in clinical settings.

## 3 Conclusion

In this study, we propose a novel transfer learning‐based approach for multi‐indicator collaborative optimization, enabling the efficient design of meta‐biosensors. Our methodology leverages pre‐training on low‐dimensional, simple structures to capture general design patterns, and subsequently fine‐tuning on high‐dimensional, complex structures via transfer learning. This strategy reduces reliance on large‐scale datasets by approximately 50% while enhancing design efficiency and overall performance. Unlike traditional single‐indicator optimization methods, we achieve the first comprehensive optimization of multiple performance indicators, including the Q factor, FoM, and ESA, thereby bridging the research gap in multi‐indicator optimization for meta‐biosensors. Furthermore, we quantify the relative contributions of these indicators to the overall performance, with the Q factor accounting for 26.09%, FoM for 48.42%, and ESA for 25.49%. Quantifying the contributions of FoM, Q factor, and ESA to frequency shift enables a more targeted optimization of biosensor designs, which helps guide design priorities and improve computational efficiency by reducing unnecessary simulations and provides a reliable reference for selecting the best optimization strategy based on specific application needs, ultimately enhancing overall performance. Both experimental and simulation validation results demonstrate that the meta‐biosensors designed using this method exhibit superior performance, achieving a pg‐level detection limit for biological marker molecules with remarkable sensitivity and reliability. This innovative approach not only provides an effective solution for trace biomolecule detection but also establishes an efficient optimization framework for meta‐biosensor design, which significantly reduces computational costs and data requirements. Moreover, the methodology can be extended to other multi‐indicator optimization tasks beyond biosensing applications. Our work will accelerate the development of intelligent sensors and optoelectronic devices and drive the design and application of next‐generation smart materials.

## 4 Experimental Section

### Dataset Collection and Division

In this study, electromagnetic simulations were performed using COMSOL Multiphysics (version 5.4) software to generate the metasurface structure and its spectral response dataset. The metasurface structure consists of a chain of resonators with two adjustable opening widths (G1 and G2), arranged in a rectangular lattice. The resonators were made from metal (gold), and the substrate material was quartz. The designed metasurface model was shown in Figure 1e. The specific geometric parameters were as follows: the periods in the x and y directions were Px = 37 µm and Py = 74 µm; the substrate thickness was T = 500 µm; the resonator length was L = 30 µm, width was W = 5 µm, resonator gap was S = 7 µm, and G1 and G2 were the tunable opening widths to manipulate the interference coupling between electric quadrupole and magnetic dipole (see Figure S10, Supporting Information for details). The simulation range covers the transmission spectra in the 1–3 THz frequency band. By systematically scanning different combinations of G1 and G2 (ranging from 2 to 15 µm, with a step size of 0.1 µm), a total of 17161 data sets were generated (see Figure S11, Supporting Information for details). In the pre‐training phase, 131 data sets were randomly selected from the low‐dimensional simple model (with G1 fixed and G2 adjustable), and the dataset was divided into training and validation sets in an 80:20 ratio to capture the basic spectral response patterns of the metasurface. In the transfer learning phase, the combined effects of G1 and G2 were learned, using 50% of the data, which was divided into training and validation sets with an 80:20 ratio. The remaining 50% was used for performance comparison experiments.

### Network Architecture

This study employs a deep neural network model based on transfer learning for the inverse design of meta‐biosensors. The model consists of two parts: a forward modeling neural network (FNN) and an inverse modeling neural network (INN), which were jointly trained and optimized in a tandem configuration. During the training process, the INN takes the target spectral response and performance indicators as input and predicts the corresponding structural design parameters. These predicted parameters were then passed into the FNN, which generates the spectral response based on them. Both INN and FNN were optimized simultaneously through backpropagation of the comprehensive loss function. Gradients were backpropagated through the entire network, allowing both INN and FNN to adjust their parameters together. This joint training approach enables the network to learn both the forward and inverse mappings concurrently, effectively overcoming the non‐uniqueness problem in inverse problems. Specifically, the entire cascaded network using a comprehensive loss function composed of two components were optimized: the INN loss, which measures the mean square error between the predicted structural parameters of INN and the actual structural parameters. And the FNN loss, which measures the mean square error between the spectral response and related performance indicators (Q factor, FoM, ESA) predicted by FNN and the simulation results. The specific formulas were as follows:

(1) $$loss_{INN}=\frac{1}{N_{1}}\;\sum_{i=0}^{N_{1}}{\left(G_{pred}-G_{sim}\right)}^{2}$$

(2) $$loss_{FNN}=\frac{1}{N_{2}+N_{3}}\;\left(\sum_{i=0}^{N_{2}}{\left(S_{pred}-S_{sim}\right)}^{2}+\sum_{i=0}^{N_{3}}{\left(E_{pred}-E_{sim}\right)}^{2}\right)$$

here, *G_pred_* and *G_sim_* represent the structural parameters of simulation and model prediction, respectively, and *N*_1_ was the number of tunable structures. *S_pred_* and *S_sim_* represent the transmission coefficients of simulation and model prediction at each frequency point, respectively, and *N*_2_ was the number of frequency points. *E_pred_* and *E_sim_* represent the performance evaluation indicators of simulation and model prediction, and *N*_3_ was the number of evaluation indices.

### Transfer Learning Strategy

The transfer learning strategy was divided into two stages: pre‐training and fine‐tuning. The pre‐training phase starts by using a relatively simple low‐dimensional dataset to pre‐train the model and learn the general relationship between metasurface structural parameters and spectral responses. This phase uses a dataset with fewer structural parameters (only adjusting G_2_ while fixing G_1_). During the pre‐training phase, the network learns general features of metasurface design and spectral responses. The fine‐tuning phase transfers the knowledge learned from the low‐dimensional model to the design of a more complex high‐dimensional model using transfer learning. The weights and biases of the pre‐trained model were used as initial parameters for fine‐tuning the model. The model on a high‐dimensional dataset was fine‐tune d, adjusting the weights and biases of the network.

### Network Parameters

The FNN consists of six layers, including one input layer, four hidden layers, and one output layer. The number of neurons in each layer was (2, 256, 256, 256, 256, 333). The ReLU activation function for all hidden layers were employed to enhance non‐linearity and improve training stability. The INN consists of seven layers, including one input layer, five hidden layers, and one output layer. The number of neurons in each layer was (333, 256, 256, 256, 256, 256, 2). Similarly, the ReLU activation function was used for all hidden layers. In this model, the batch size was set to 64, and the Adam optimizer was used in the optimization process. In this experiments, the initial learning rate was set to 0.0001, and the model was trained for 10000 epochs.

### Computational Environment

The deep learning experiments in this paper were implemented on a computer equipped with an Intel Core i9‐13900K CPU (3.00 GHz), and an NVIDIA GeForce RTX 4090 GPU. The operating system used was Microsoft Windows. The deep learning process was based on the PyTorch framework.

### THz Measurement

The transmission spectra of the samples were measured using a commercial terahertz spectrometer (Advantest TAS7400). The system operates within a frequency range of 0.5 to 4 THz, with a spectral resolution of approximately 7.6 GHz. To suppress water vapor interference, all of this tests were conducted at room temperature (23 °C) with a relative humidity of less than 3%. To ensure the robustness of the results, all samples were tested multiple times, and the Fourier transform was applied to obtain the frequency‐domain signals. The terahertz transmission signals of the bare metasurface and samples were measured with different concentrations of homocysteine (Hcy) solution.

## Conflict of Interest

The authors declare no conflict of interest.

## Author Contributions

S.W. performed investigation, methodology, software, data analysis, wrote‐the original draft and edited the original draft. B.L. performed investigation, experiment, original draft. X.W. performed data analysis. Z.J. performed data analysis. S.C. performed data analysis. Y.Z. performed funding acquisition. L.Z. performed funding acquisition, supervision. Y.P. performed funding acquisition, supervision, wrote – review and edited the original draft. S.W. and B.L. were Co‐first author.

## Supporting information



Supporting Information

## Data Availability

The data that support the findings of this study are available from the corresponding author upon reasonable request.
