# Supplementary material for: Transfer Learning Empowered Multiple‐Indicator Optimization Design for Terahertz Quasi‐Bound State in the Continuum Biosensors
Source: Adv Sci (Weinh). 2025 Apr 27;12(27):2504855. doi: 10.1002/advs.202504855 (PMC12279224; doi:10.1002/advs.202504855)
Supplement: Supplementary file 1 — Supporting Information [file ADVS-12-2504855-s001.docx]

**Supporting Information**

**Transfer Learning Empowered Multiple-Indicator Optimization Design for Terahertz Quasi-Bound State in the Continuum Biosensors**

Shengfeng Wang**^1, #^,** Bingwei Liu**^1, #^**, Xu Wu^1^, Zuanming Jin^1^, Yiming Zhu^1,3,§^, Linjie Zhang^2,£^, Yan Peng**^1,3,*^**

^1^Shidong Hospital Affiliated to University of Shanghai for Science and Technology, Terahertz Technology Innovation Research Institute, Shanghai Key Lab of Modern Optical System, Shanghai Institute of Intelligent Science and Technology, University of Shanghai for Science and Technology, Shanghai, China

^2^State Key Laboratory of Quantum Optics and Quantum Optics Devices, Institute of Laser Spectroscopy, Shanxi University, Taiyuan, Shanxi, China

^3^Shanghai Institute of Intelligent Science and Technology, Tongji University, Shanghai, China

^#^ Co-first author

Corresponding author: ^*^py@usst.edu.cn, ^£^zlj@sxu.edu.cn, ^§^ymzhu@usst.edu.cn

# S1. The supplementary evaluations of the model design capability and spectral reconstruction ability.

To further validate the generalization and adaptability of our proposed tandem neural network framework, we present two additional design cases in Figure S1, beyond the example shown in Figure 2 of the manuscript. These cases aim to demonstrate the effectiveness of our method under varying design requirements.

Compared with the original case, the two new examples differ in the following ways. For the INN, the input target spectra are different, requiring the network to design different meta-biosensor structures. For the FNN, the input structural parameters are different, requiring the network to predict the corresponding spectral responses of different meta-biosensors.

Figure S1 illustrates two inverse design cases with significantly different target spectra in resonance features and line shapes. For each case, we evaluate the INN and FNN model performance at four training stages. As training progresses, both the structural design error and spectral reconstruction loss decrease noticeably. These results demonstrate that the proposed model achieves high accuracy across diverse design conditions, confirming its generalization capability and overall effectiveness.

**

**

**Figure S1. a, b.** Evolution of model performance at different training epochs (100, 300, 500, and 5000). Predicted structures (red) are compared with ground truth structures (blue) to assess INN accuracy. The transmission spectra predicted by FNN (dashed red) are compared with COMSOL simulation results (blue) to assess FNN accuracy. The shaded grey area indicates the prediction loss.

# S2. The process of entire optimization design.

**STEP1.** Pre-training the network model: Train the tandem neural network, which consists of INN and FNN, using a dataset of metasurface structures and their corresponding spectral responses.

**STEP2.** Learning the structure–performance mapping through transfer learning: Fine-tune the pre-trained model to establish the relationship between metasurface sensor structures and their spectral responses and performance evaluation indicators (Q factor, FoM, ESA).

**STEP3.** Predicting spectral responses and performance indicators: Utilize the trained network to predict spectral responses and key performance indicators (Q factor, FoM, ESA) for biosensors with different structural parameters.

**STEP4.** Multi-indicator optimization: Use frequency shift as a unified optimization criterion to systematically adjust biosensor structures and analyze the contribution of each performance indicator.

# S3. The calculation of Q, FoM, and effective sensing area.

We utilized a standard Fano lineshape equation to fit the transmission spectra and thereby determine the Q factors. The formula is as follows:

| $T_{Fano}=\left\vert a_{1}+ja_{2}+\frac{b}{\omega-\omega_{0}+j\gamma} \right\vert$ | (1) |
| --- | --- |

Here, $a_{1}$, $a_{2}$and $b$ represent real constant coefficients, while $\omega_{0}$ denotes the resonance frequency and $\gamma$ signifies the damping rate. The Q factor reflects the quality factor of the sensor, which can be derived from the formula $Q=\omega_{0}/(2\gamma).$

Figure of Merit (FoM) is used to measure the comprehensive performance of sensors between resolution and signal strength, defined as:

| $FoM=Q*I$ | (2) |
| --- | --- |

Here,$Q$ is the quality factor of the sensor, $I$ is the peak transmission intensity.


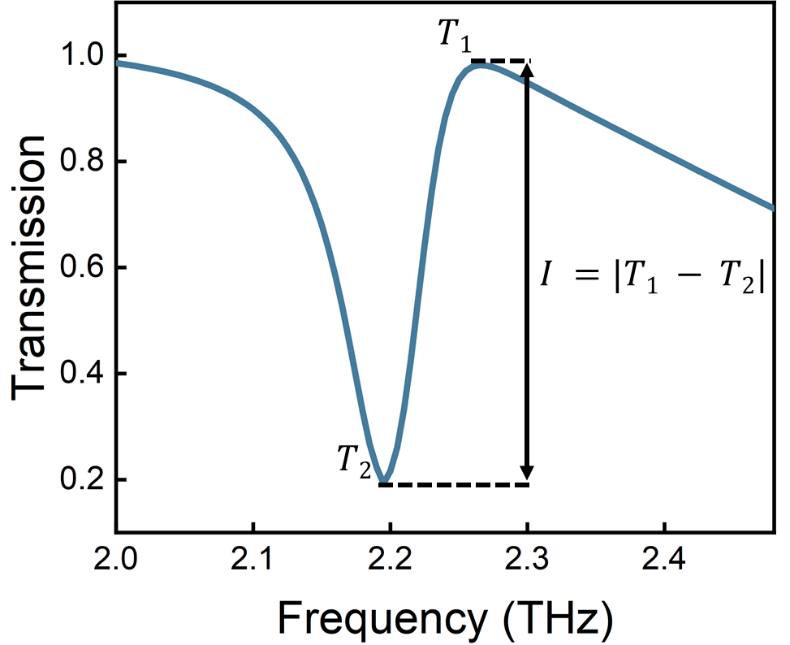


**Figure S2.** Calculation method for the peak transmission intensity.

At this stage, the effective sensor area (ESA) values are obtained through COMSOL simulations. The ESA quantifies the portion of the sensing region where the electric field is sufficiently strong to interact effectively with target analytes. Its calculation is based on the spatial distribution of the electric field intensity obtained by COMSOL simulation, with a defined threshold to determine the boundaries of effective interaction. The resonant cavity regions corresponding to the QBIC resonance mode excited by meta-biosensors are Area1, Area2, and Area3. Therefore, the overall sensing region is divided into these three rectangular subregions. To reduce computational complexity, we assume that the electric field is approximately uniform in the longitudinal direction of each rectangular subregion. Based on this assumption, we calculate ESA by analyzing the transverse electric field distribution along a representative cross-sectional line perpendicular to the longitudinal axis of each subregion. In each subregion, the maximum value of the electric field intensity is first identified. The effective boundary is then defined as the position at which the field intensity decays to 1/e of this maximum value. This threshold corresponds to a conventional decay limit, capturing the spatial extent of the strong-field region. The transverse electric field distribution of the three sub-regions is shown in Figure S3 (b-d). The portion of this profile where the field intensity remains above the 1/e threshold defines the effective width. Multiplying this width by the fixed longitudinal height of the subregion yields the effective area for that region. The total ESA is obtained by summing the effective areas of the three subregions.

It is important to note that this physically defined ESA is only solely during the training data generation stage to provide accurate labels for the neural network. Once the FNN is trained, it can directly predict ESA from structural parameters, bypassing the need for electric field calculations or spectral simulations.


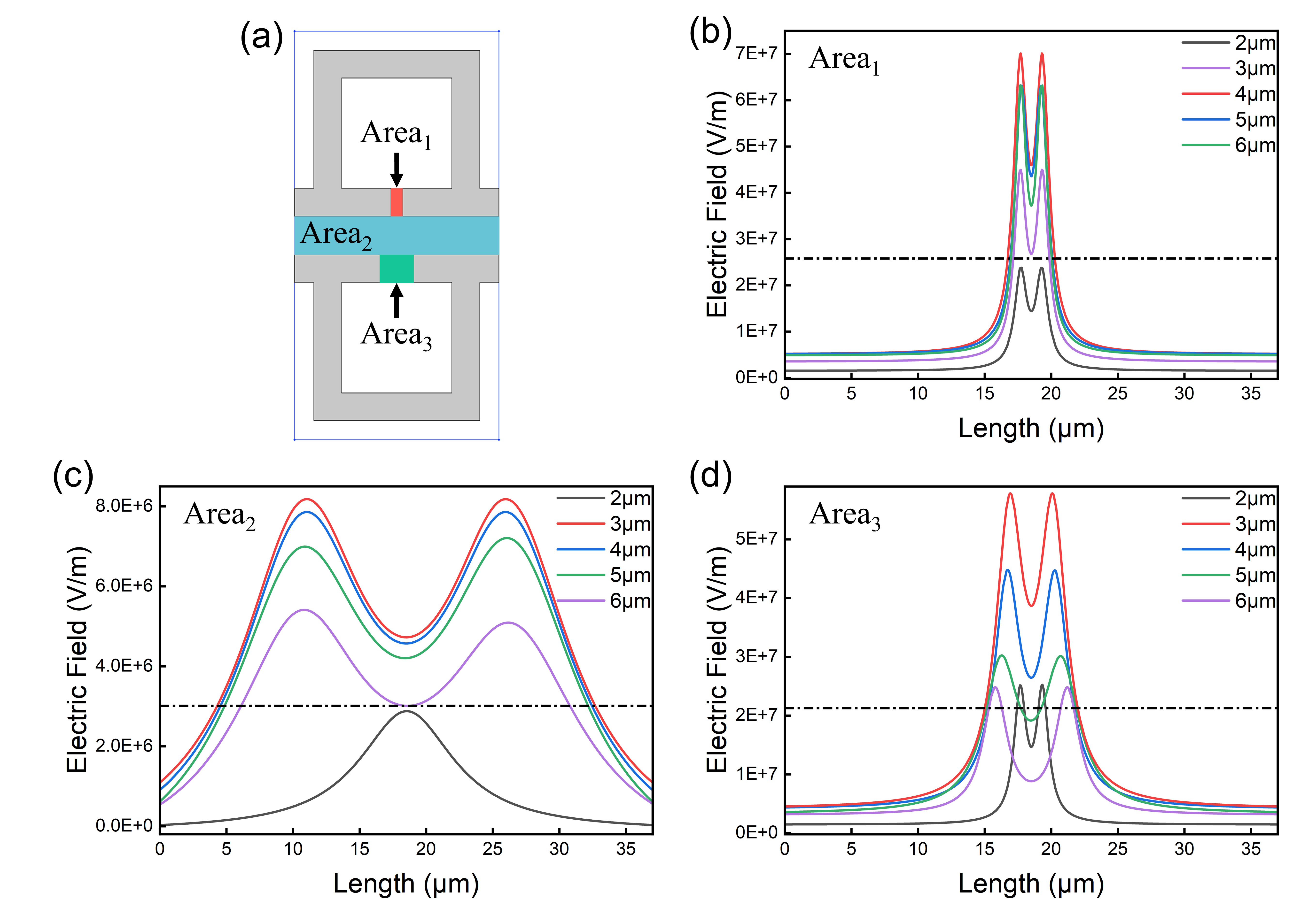


**Figure S3.** Calculation method for the effective sensor Area. **a.** The ESA is divided into three regions: Area_1_, Area_2_, and Area_3_. **b-d.** Approximate calculation of the effective sensor area for the three regions under different gap widths G_2_.

# S4. The calculation of the frequency shift.

The frequency shift is a fundamental indicator for evaluating the performance of biosensors, as it directly reflects the sensor’s response to variations in the surrounding biological environment. First, when biomolecules bind to the sensor surface, they induce changes in the local refractive index, leading to a shift in the resonance frequency of the system. This measurable shift provides a direct assessment of the ability of meta-biosensors to detect and quantify target analytes. Second, frequency shift is closely related to sensitivity. A larger frequency shift at a given refractive index change indicates higher sensitivity, which is crucial for detecting low-concentration biomolecules.

Therefore, we adopt frequency shift as the primary performance metric to ensure that the designed metasurface biosensor not only achieves comprehensive optimization in terms of Q factor, FoM, and ESA but also delivers optimal performance in biosensing efficacy. We simulated the frequency shift caused by biological samples using COMSOL Multiphysics (version 5.4). In the simulation, we analyzed ultrathin analytes with a fixed refractive index of 1.5 RIU and different concentrations, which fully covered the metasurface unit, and calculated the frequency shift ∆f induced compared with the bare chip. We approximately assume that the biological samples are uniformly spread over the surface of the metasurface biosensor and simulate using a thickness of 5 μm.

# S5. The calculation of the contribution of multiple performance indicators to frequency shift.

Random Forest (RF) is an ensemble learning method that primarily constructs multiple decision trees using the Bootstrap aggregation (Bagging) approach, and outputs predictions through voting or averaging. Its main advantages include robustness to high-dimensional data, excellent generalization ability, and the natural ability to evaluate feature importance. To quantify the specific contributions of Q factor, FoM, and ESA to frequency shift, we employed the Random Forest method for a detailed analysis of these three indicators. The specific steps are as follows: First, Q factor, FoM, and ESA were used as input features, with frequency shift as the target variable, to construct the complete dataset. Then, this dataset was used to train a Random Forest regression model. During the model training process, we selected the Bagging (Bootstrap aggregation) method, which generates multiple training subsets by randomly sampling with replacement from the training set. A decision tree is trained independently on each subset, and the final prediction result is the average of the weak learner outputs. At last, to quantify the relative contributions of each feature to the frequency shift, we calculated the relative importance scores of each feature based on the reduction of impurity at the split nodes of each decision tree. These scores reflect the contribution of each feature to the reduction in mean squared error (MSE) during model training, indirectly revealing the influence of different features on the frequency shift prediction. By calculating the root mean square error (RMSE) of the cross-validation, we quantified the deviation between the model's predicted values and the true values and further evaluated the model's stability and accuracy. The analysis results show that the relative contributions of Q factor, FoM, and ESA to frequency shift are 26.09%, 48.42%, and 25.49%, respectively.

# S6. Quantifying the contributions of performance indicators in other devices using our model.

To further demonstrate the generalizability of our proposed model, we applied the same framework to the design of other metasurfaces. As shown in Figure S4, the contribution ratios were clarified (52.58% for FoM, 30.12% for Q factor, and 17.30% for ESA). These results indicate that while the specific numerical values of the contribution ratios may vary depending on the device structure, the underlying method remains broadly applicable across different biosensor designs.

Our approach offers valuable guidance for multi-indicator optimization. By analyzing the relative contributions of performance indicators, designers can identify which metric has the greater impact on a given structure and accordingly adjust the optimization direction. This strategy not only reduces reliance on blind trial-and-error methods but also enhances design efficiency, enabling more targeted and effective optimization processes.


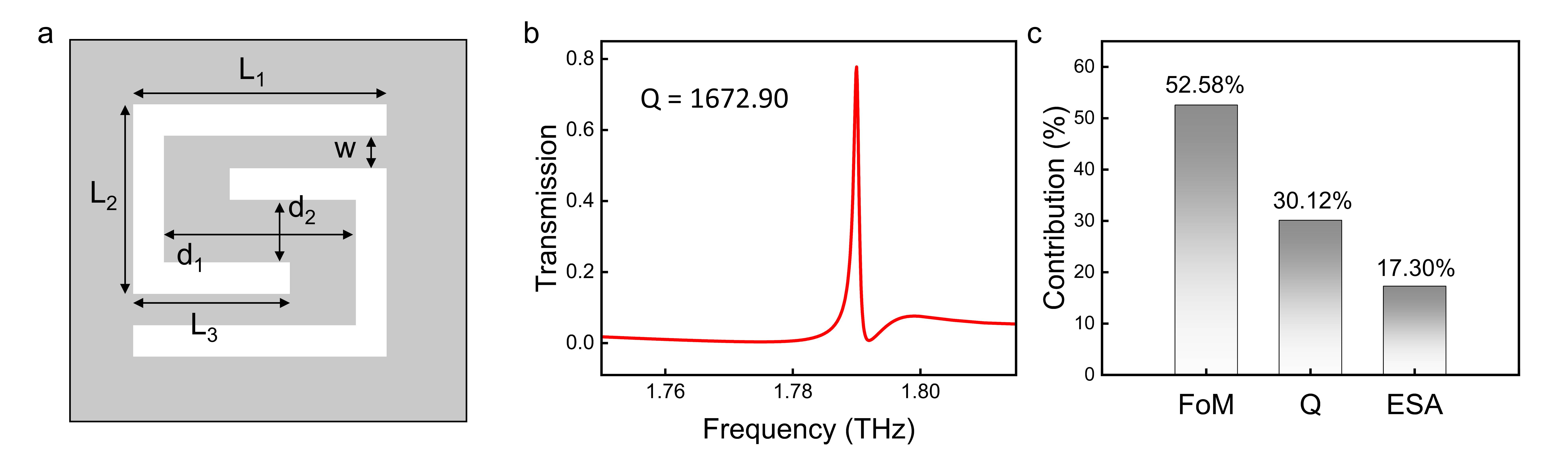


**Figure S4. a.** The structure of the newly designed metasurface. **b.** The spectral response of the metasurface. **c.** Analysis of the contribution of each evaluation indicator to sensor performance.

# S7. Fabrication and performance test of metasurface sensor.

The metasurface sensor designed by us is fabricated by depositing chromium and gold films on a double-sided polished quartz substrate and then patterning by conventional lithography technology. Here, chromium is used as an adhesion layer. We have processed a series of chips to study the transmission characteristics of the metasurface sensor in the terahertz band under laboratory conditions. The test equipment is a terahertz time-domain spectrometer (THz-TDS), which can accurately measure the transmittance of the sample at different frequencies. The test steps are as follows: 1. Sample fixation: The processed metasurface sensor is fixed on the sample frame of the terahertz time-domain spectrometer to ensure that the terahertz wave is incident on the sample surface. Light source setting: The excitation source in the terahertz band is set to a scanning frequency range of 0.1 THz to 3.0 THz, covering the working range of the resonant frequency in the design structure. Data acquisition: Through the THz-TDS measurement system, the transmission and reflection spectra of the metasurface sensor in the terahertz band are collected, with special attention to the resonance frequency and the sensitivity of the sensor.

# S8. Analysis of Reasons for Performance Degradation.

It is well known that the experimental value of the Q factor is usually lower than the simulated value, so this measurement result does not reach the optimal state. Our research suggests that there may be many factors that jointly cause the degeneration of the Q factor: (1) Non-vertical incident terahertz waves will cause the intensity attenuation and broadening of QBIC resonance, and we also found the influence of additional BIC states excited by angular asymmetry; (2) The influence of polarization angle will make QBIC drowned in the background; (3) The manufacturing tolerance of micro-nano fabrication technology tends to show a Gaussian distribution, thus affecting the robustness of resonance; (4) Sample materials, rough surfaces and impurities will lead to inevitable Ohmic, scattering and impurity losses; (5) The resolution ratio limitation of the terahertz spectrometer used cannot observe the QBIC lineshape that should have been narrower.

# S9. Preparation of biomarker samples for sensing performance test.

To validate the sensing performance of the designed metasurface, we prepared homocysteine (Hcy) solutions of varying concentrations as the target for detection, as shown in Table S1. In our previous work, we have shown that homocysteine does not hydrolyze or bind with water molecules in an aqueous solution, and its properties remain stable^[1, 2]^. Pure homocysteine (≥95%, CAS: 454-29-5) was obtained from Sigma-Aldrich, with a molecular weight of 135.18 g/mol. Here, we used ultrapure water as the solvent, weighed 13.5 mg of pure homocysteine powder, dissolved it in 1 mL of ultrapure water, and mixed it thoroughly using a vortex mixer (Huxi vortex-2), obtaining a 0.1 mmol/mL homocysteine aqueous solution. Then we prepared 6 different concentrations of Hcy solutions by diluting the 0.1 mmol/mL Hcy solution with ultrapure water. In the experiment, we used a pipette to take 20 μL of each Hcy solution and fixed the height of the pipette to ensure consistent area coverage on the metasurface. We then pretreated the sample using the drying mode of a biological concentrator (Eppendorf concentrator plus) to ensure the sample molecules were evenly distributed on the metasurface. Before each test, we cleaned the transfer surface three times with phosphate-buffered saline and then dried it with blotting paper to reduce interference.

**Table S1.** Prepared 6 groups of Hcy aqueous solution with different concentrations and the measured content.

| **Number** | **Sample concentration**  **(ng/μL)** | **Sample volume**  **(μL)** | **Sample mass**  **(ng)** |
| --- | --- | --- | --- |
| 1 | 1.68975 | 20 | 33.795 |
| 2 | 6.759 | 20 | 135.18 |
| 3 | 67.59 | 20 | 1351.8 |
| 4 | 337.95 | 20 | 6759 |
| 5 | 1351.8 | 20 | 27036 |
| 6 | 5407.2 | 20 | 108144 |

# S10. Multipolar Decomposition

To quantitatively analyze the dominant components of QBIC resonance., we calculate the scattering cross sections of metasurface by the the multipolar decomposition method. The Cartesian multipolar decomposition was carried out in this work, in which the polar moment is obtained by integrating the carrier density $\rho\left( r \right)$ or the current density $J\left( r \right)$ Within the unit structure, and then calculate the sum of the far-field scattered power of all polar moments at each frequency point, and finally obtain the total far-field scattered power ^[^[^3-5^](file:///D:\Desktop\论文\小论文\8.刘\1\ScienceDirect_files_27Aug2024_05-28-07.147\1-s2.0-S1385894723010781-mmc1.docx#_ENREF_3)^]^.

The computing formulas of each polar moment are as follows, where c is the speed of light, $\omega$ is the frequency of light, $\alpha$ and $\beta$ represents the x-, y- and z-direction of the coordinate axis:

Electric dipole moment：

| $P=\frac{1}{i\omega}\int Jd^{3}r$ | (3) |
| --- | --- |

Magnetic dipole moment：

| $M=\frac{1}{2c}\int\left( r\times J \right)d^{3}r$ | (4) |
| --- | --- |

Toroidal dipole moment：

| $T=\frac{1}{10c}\int\left[ \left( r\cdot J \right)r-2r^{2}J \right]d^{3}r$ | (5) |
| --- | --- |

Electric quadrupole moment：

| $Q_{\alpha\beta}=\frac{1}{2i\omega}\int\left[ r_{\alpha}J_{\beta}+r_{\beta}J_{\alpha}-\frac{2}{3}\left( r\cdot J \right) \right]d^{3}r$ | (6) |
| --- | --- |

Magnetic quadrupole moment：

| $M_{\alpha\beta}=\frac{1}{3c}\int\left[ \left( r\times J \right)_{\alpha}r_{\beta}+\left( r\times J \right)_{\beta}r_{\alpha} \right]d^{3}r$ | (7) |
| --- | --- |

The computing formulas of the far-field scattered power corresponding to each polar moment are as follows:

Electric dipole：

| $\mathrm{ED}=\frac{2\omega^{4}}{3c^{3}}\left\vert P \right\vert^{2}$ | (8) |
| --- | --- |

Magnetic dipole：

| $MD=\frac{2\omega^{4}}{3c^{3}}\left\vert M \right\vert^{2}$ | (9) |
| --- | --- |

Toroidal dipole：

| $\mathrm{TD}=\frac{2\omega^{6}}{3c^{5}}\left\vert T \right\vert^{2}I_{Q}^{e}$ | (10) |
| --- | --- |

Electric quadrupole：

| $\mathrm{EQ}=\frac{\omega^{6}}{5c^{5}}\left\vert Q_{\alpha\beta} \right\vert^{2}$ | (11) |
| --- | --- |

Magnetic quadrupole：

| $\mathrm{MQ}=\frac{\omega^{6}}{40c^{5}}\left\vert M_{\alpha\beta} \right\vert^{2}$ | (12) |
| --- | --- |

The computing formula of the total far-field scattered power is as follows:

| $I=\frac{2\omega^{4}}{3c^{3}}\left\vert P \right\vert^{2}+\frac{2\omega^{4}}{3c^{3}}\left\vert M \right\vert^{2}+\frac{2\omega^{6}}{3c^{5}}\left\vert T \right\vert^{2}+\frac{\omega^{6}}{5c^{5}}\left\vert Q_{\alpha\beta} \right\vert^{2}+\frac{\omega^{6}}{40c^{5}}\left\vert M_{\alpha\beta} \right\vert^{2}$ | (13) |
| --- | --- |


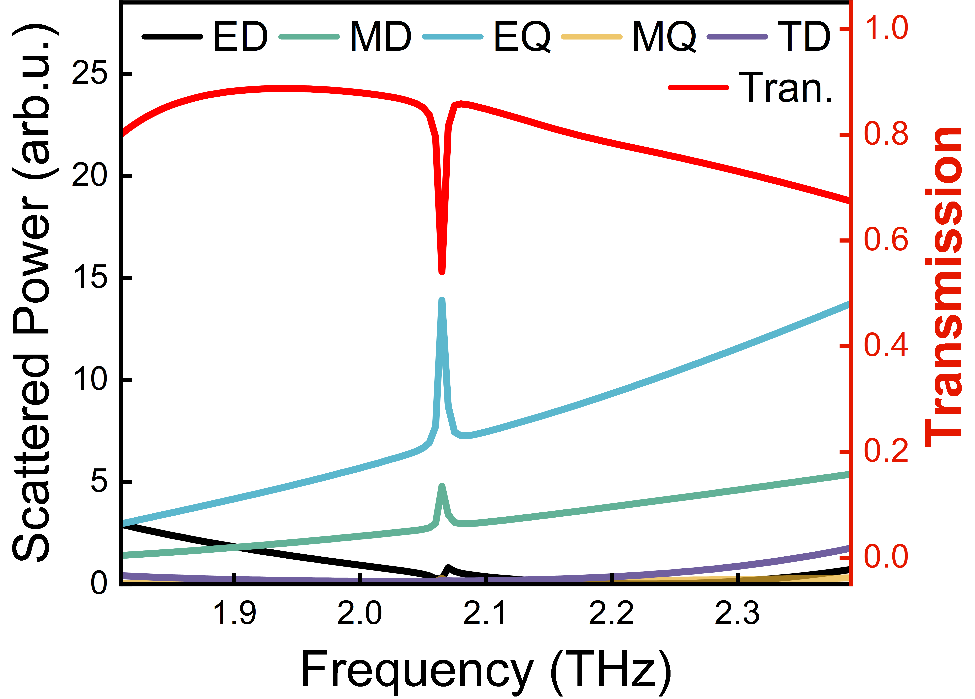


**Figure S5.** Multipolar decomposition results for the far-field scattered power of the metasurface biosensor corresponding to α = 0 under x-polarized terahertz waves.

# S11. Data Collection for Deep Learning

We randomly collect data byCOMSOL Multiphysics (version 5.4) using a desktop (Windows 10 operation system, GeForce GTX 4090 GPU, Intel Core i9-13900K CPU (3.00 GHz), and 128GB of RAM). The detailed structure parameter range is provided in Table S2.

**Table S2.** Structure parameters of the meta-biosensor.

| **Structure** | **Value** | **Unit** |
| --- | --- | --- |
| G_1_ | 2.0 to 15.0 | micron |
| G_2_ | 2.0 to 15.0 | micron |
| P_x_ | 37 | micron |
| P_y_ | 74 | micron |
| L | 30 | micron |
| W | 5 | micron |
| S | 7 | micron |
| T | 500 | micron |

**Reference**

[1] T. Li, H. Ma, Y. Peng, X. Chen, Z. Zhu, X. Wu, T. Kou, B. Song, S. Guo, L. Liu, Y. Zhu, Gaussian numerical analysis and terahertz spectroscopic measurement of homocysteine, Biomed Opt Express 9(11) (2018) 5467-5476.

[2] L. Wang, X. Wu, Y. Peng, Q. Yang, X. Chen, W. Wu, Y. Zhu, S. Zhuang, Quantitative analysis of homocysteine in liquid by terahertz spectroscopy, Biomed Opt Express 11(5) (2020) 2570-2577.

[3] P.C. Wu, C.Y. Liao, V. Savinov, T.L. Chung, W.T. Chen, Y.W. Huang, P.R. Wu, Y.H. Chen, A.Q. Liu, N.I. Zheludev, D.P. Tsai, Optical Anapole Metamaterial, ACS Nano 12(2) (2018) 1920-1927.

[4] B. Yang, W. Liu, Z. Li, H. Cheng, D.Y. Choi, S. Chen, J. Tian, Ultrahighly Saturated Structural Colors Enhanced by Multipolar-Modulated Metasurfaces, Nano Lett 19(7) (2019) 4221-4228.

[5] L. Zhang, K. Gao, F. Lu, L. Xu, M. Rahmani, L. Sun, F. Gao, W. Zhang, T. Mei, Visible-Band Chiroptical Meta-devices with Phase-Change Adjusted Optical Chirality, Nano Lett 22(18) (2022) 7628-7635.
